# Effects of migrant workers’ health capital on their returning home intentions: evidence from China

**DOI:** 10.3389/fpubh.2025.1473435

**Published:** 2025-02-03

**Authors:** Haitao Li, Haibo Li, Hao Xiang

**Affiliations:** ^1^School of Administration and Law, Hunan Agricultural University, Changsha, China; ^2^School of Business, Hunan International Economics University, Changsha, China; ^3^School of Economics and Management, Changsha University, Changsha, China; ^4^School of Elevator Engineering, Shaoyang Polytechnic, Shaoyang, China

**Keywords:** health capital, returning home intentions, migrant workers, social capital, economic capital

## Abstract

**Background:**

The health problems and migration problems of China’s huge migrant workers are related to the implementation effect of new-type urbanization and rural revitalization strategy. A thorough examination of the effect of migrant workers’ health on their returning home intentions and its mechanism is imperative.

**Objectives:**

This study aims to understand the influence of migrant workers’ health capital (HC) in their returning home intentions (RHI). For this, using capital theory, this study developed an empirical model to probe the impact of Chinese migrant workers’ health on their RHI and analyze the paths that this influence may follow to form the RHI of migrant workers.

**Methods:**

A theoretical model was established from the perspective of capital to study the relationship between health and migrant workers’ migration. A probit model was used to probe the impact of Chinese migrant workers’ HC on their RHI, and propensity score matching (PSM), replacing the explained variable (REV), and transforming the model (TM) were used for the robustness test. Stepwise coefficient test (SCT) and Sobel test (ST) were used to analyze the mediating effects of economic capital (EC) and social capital (SC) in the relationship of HC affecting RHI. Data were collected from a sample of 810 migrant workers from three provinces located in eastern, central, and western China using a survey questionnaire.

**Results:**

Migrant workers’ HC had a significant and negative impact on their RHI, and mental health (MH) had a greater impact on migrant workers’ RHI than physical health (PH). There is regional heterogeneity in the effect of HC on the RHI of migrant workers in China. In addition, economic capital (EC) mediated indirectly the relationship between HC and RHI, while the mediating role of social capital in the relationship between HC and RHI was not significant.

**Conclusion:**

Migrant workers’ HC is an important factor affecting the formation of their RHI, in which EC plays an intermediary role. This study extends and deepens our understanding of HC and helps to understand the role of health in shaping RHI from the perspective of capital, and the finding confirms the validity of the “salmon bias effect” hypothesis in the Chinese context. These findings can also help local government pay more attention to returning migrant workers’ HC.

## Background

China’s long-term urban–rural dual structure leads to the concentration of innovation resources in cities, which promotes the rapid development of China’s urbanization, and restricts the innovation vitality and economic development of rural areas because it leads to the hollowing out of various resources in rural areas (51). China put forward a poverty alleviation strategy in 2015 and a rural revitalization strategy in 2017 to promote the development of rural areas. Talent is the key to implementing these strategies. Migrant workers returning home is needed for rural revitalization, which can provide human and intellectual support for rural revitalization (1). Moreover, migrant workers’ returning home has a “return effect” (2), which can bring multiple compensations of capital, information, and technology for rural development, thus promoting the development of the rural economy (3). Therefore, it is of great practical significance for migrant workers to return home.

Previous studies have proved that migration is influenced by a variety of factors. As an important part of human capital, health is considered to be one of the important screening mechanisms for individuals in the migration process and is a key factor influencing the migration of mobile populations. There are two classic theories about the health of international migrants: The Healthy Migrant Hypothesis and the Salmon Bias Hypothesis. The former emphasizes that people in good health have a stronger desire to migrate (4), whereas the latter emphasizes that migrants in poor health are more inclined to return to or stay close to their place of origin (5). Both explain the relationship between health and migration from different perspectives, and both have been empirically supported in the context of international migration (6). In China, both the health problems and migration issues of migrant workers are receiving more and more attention and concern from governments, but research on the relationship between health and returning home intentions is still lacking. For Chinese migrant workers, the following questions need to be further explored: (1) whether health affects their returning home intentions? (2) does the migration of Chinese migrant workers also have a “salmon bias” effect? and (3) how does health affect their returning home intentions?

This study aims to understand the influence of migrant workers’ health capital (HC) in their returning home intentions (RHI). For this, based on the perspective of capital theory, this study developed an empirical model to probe the impact of migrant workers’ health on their returning home intentions and analyze the paths that this influence may follow to form the RHI of migrant workers. The data used for empirical analysis were collected from a sample of 810 migrant workers from three provinces located in eastern, central, and western China. This study helps to extend and deepen our understanding of migrant workers’ HC and its role in shaping their RHI and provide Chinese evidence for the “salmon bias effect” hypothesis. This study can also provide a basis for the government’s policy on migrant workers’ health and migration.

The rest of the article is structured as follows: The following section is a literature review, and the third describes the theoretical framework and sets out the hypotheses. The fourth introduces the methodological design of the empirical work, and the fifth reports the main empirical results. The sixth section presents a discussion of the results, and the final outlines the conclusion and their implications for policy and further research.

## Literature review

### Study on the factors influencing the migrant workers’ RHI

According to previous research, the factors affecting migrant workers’ RHI can be categorized into two levels: macro and micro factors. At the macro level, the early push–pull theory and dual structure theory focus on the perspective of macroeconomics, environment, and other social structures and believe that regional differences are the main reason for population migration, in which the impact of housing has received extensive attention from scholars, and scholars generally believe that the housing prices of the city have a significant impact on the flow of population (7–10). The regional environment also has a significant impact on the decision of migrant workers’ returning (11, 12). The labor market segmentation theory suggests that migrants return home because they are unable to enter the job market in the city of importation, as local domicile is a prerequisite for certain specific jobs (13). Some studies have found that the household registration system and urban settlement threshold are important factors leading to the return (14, 15).

At the micro level, the relevant theories and empirical studies mainly examine population migration in the changing process of the life cycle of individuals and their families and reveal the law of its development and change with the occurrence of different life events (16). Based on the rational person hypothesis, the individual characteristics of migrants such as age and income level affect their migration (17). Some studies show that individual characteristics of rural labor such as education level, age, gender, and marital status significantly affect their return intentions (18, 19). The new migration economic theory, based on the principle of maximizing the expected income and minimizing the risk of the family, believes that family factors have an impact on population migration (20). Ren and Shi (2) found that family factors such as conjugal reunion, child-rearing, and supporting parents affect the return migration of rural laborers. Network theory emphasizes the influence of social networks on the return of migrant workers and believes that social network plays a crucial role in the process of the migrant population’s adaptation and integration into local society (21–23).

### Study on the relationship between health and population migration

Migration and health are closely linked. In international academic research on immigrant health, there is a well-known “Hispanic Paradox”: Despite their lower socioeconomic status and limited access to health care, Latino immigrants in the United States generally have better health than native-born Americans (5, 24). Two theories explain this paradox. One is the Healthy Migrant Hypothesis, which suggests that healthier workers among international migrants have a stronger desire and ability to migrate because they are more likely to be economically successful (4). The second is the Salmon Bias Hypothesis, which suggests that the health status of transnational migrant workers declines over time due to intense competition in the labor market, increased work–life stress, and inadequate healthcare services and that the deteriorating health status reduces their labor capacity, which results in unhealthy migrants or migrants with deteriorating health tending to return to or stay closer to their communities of origin than healthy migrants (5, 25). Both hypotheses in international migration have been supported by some studies.

Some studies have also applied the Salmon Bias Hypothesis and the Health Migration Hypothesis to the phenomenon of labor migration in China, for example, Guo and Ma (26) empirically analyzed that the health status of migrant workers showed a spatial pattern of gradual decline in health status, proving that the health migration effect has a significant impact on the labor migration phenomenon in China. Yao and Qin (6) found that healthier people are more likely to migrate and move away from their hometowns. Some studies found that rural laborers in better health were more likely to migrate out of their hometowns, while those in poorer health would choose to return in the long term (27).

The Literature Review shows that scholars have conducted rich research on the factors influencing migration, but in the Chinese context, the research is relatively lacking, and the mechanism of how health affects migrant workers’ returning is still relatively lacking, the role of health in the formation of migrant workers’ returning home intentions has not yet been paid enough attention, and the theoretical analyses and empirical cases of how health affect migrant workers’ returning home intentions need to be strengthened.

## Theoretical analysis and research hypothesis

### Migrant workers’ HC

Health is essential for individuals to achieve holistic development and is closely related to people’s wellbeing and social harmony (28). The idea of linking individual health to capital can be traced back to classical economics, starting with Mushkin's (29) view of health as an investment and Becker's (30) view of health as a part of human capital. Grossman (31), based on these views, introduced the term “health capital” as part of a model of the demand for goods in “good health.” The model views health as a “durable capital stock that produces healthy time outputs” and depreciates with age but can be invested in through medical treatment. Subsequently, social capital theory has brought health into the realm of sociological research, and Turner (32) discusses his sociological insights on health as capital on the basis of the social capital theories of scholars such as Lin and Coleman, which complements the human capital view of health as capital, and believed that social capital is closely related to health. Thus, it can be seen that health has both economic and social attributes. Health capital is a part with human capital; on the one hand, health capital can increase human capital accumulation (33) and promote economic capital accumulation (34). On the other hand, health capital also affects individuals’ social capital such as social networks and trust.

In this study, HC refers to migrant workers’ health conditions during their working in cities, including physical health and mental health.

### The relationship between HC and migrant workers’ RHI

As an important theory for studying the causes of population migration behavior, the push–pull theory has been widely used to analyze the influencing factors and mechanisms of population migration (52) and has strong explanatory power for the migration of migrant workers in China. The theory suggests that factors such as lack of basic amenities such as schools and hospitals as well as estrangement and tension in relationships and natural disasters in the original place of residence will prompt people to migrate to other areas. At the same time, factors such as better job opportunities, higher wages, better education, and health facilities attract people to migrate to that region (35). Later, Lee (36) extended the push–pull theory by adding internal factors affecting migration and systematically categorized the factors affecting migration into four categories: outflow-related factors, inflow-related factors, intervention barriers, and individual factors. Migration of migrant workers is the result of the combined effect of urban push, rural pull, individual characteristics, and intervening barrier factors.

This study focuses on the influence of individual health characteristics of migrant workers on their return migration. According to the push–pull theory, for migrant workers who migrate to cities, more job opportunities and higher wage income in cities are the important pull forces that attract them to enter cities. At the same time, the high cost, insecurity, and social exclusion of living in the city are important push forces that affect their return to their hometowns. As an important part of human capital, the health status of migrant workers will have an impact on these pulls and pushes, which in turn will affect their willingness to return.

### Indirect effects of HC on RHI through economic capital

From the perspective of human capital theory, health capital is a capital stock with a dual nature that can be both an input and an outcome of the production process. When health capital is viewed as an input, any investment made by an individual in his or her health capital will be treated as an asset that earns a material return through the production of healthy time (31). Therefore, as part of human capital, health is an important form of capital that people can use and invest in (31) and can be considered a personal resource that may give rise to social advantages and disadvantages (37). Previous studies have shown that health capital plays a positive role in increasing the accumulation of human capital (33) and promoting the accumulation of economic capital (34). The impact of health capital on the employability and income level of workers has been generally recognized by scholars (38, 39), rural laborers with poorer health status lack competitiveness in the job market and have difficulty in finding relatively desirable jobs (22), and the average annual income from labor only reaches 63% of that of migrant workers in better health (39). Migrant workers migrate from rural to urban areas because they have better employment opportunities and higher economic incomes than those in rural areas and may choose to return home once they are unable to find jobs that match their expectations and earn incomes that are in line with their expectations. Thus, health capital affects the willingness of migrant workers to return by influencing economic capital.

### Indirect effects of HC on RHI through social capital

According to Maslow’s hierarchy of needs theory, after satisfying basic needs such as income and housing, rural migrant workers who enter the cities will have higher-level needs such as integrating into urban society and having the same status and position as urban residents. This need is also an important factor influencing whether migrant workers choose to settle in the city or return to their hometowns for development. From the viewpoint of social capital theory, health is a reserve of biopsychosocial resources that people can utilize (40), and poor health can limit a person’s social participation, which may hinder the acquisition and accumulation of other resources (41). A large number of empirical studies have shown that health is significantly associated with social capital (42) and that good health contributes to the accumulation of urban social capital among migrant workers. Social capital is closely related to class identity, social recognition, and urban integration; the richer the social capital of migrant workers in the city, the more it helps them to gain recognition in the city and integrate into the urban society, which will reduce their willingness to leave the city and return to their hometowns. Previous studies have shown that the higher the social capital, the lower the willingness of rural laborers to return to their hometowns (43).

According to the above ways, this study believes that as a part of human capital, HC has a direct impact on migrant workers’ RHI. Meanwhile, HC can also affect migrant workers’ EC and SC and then indirectly affect their RHI. Based on this, this study proposes three hypotheses:

*H*1: HC has a direct and negative impact on migrant workers’ RHI.

*H*2: EC indirectly mediates the relationship between HC and migrant workers’ RHI.

*H*3: SC indirectly mediates the relationship between HC and migrant workers’ RHI.

**Figure 1:**
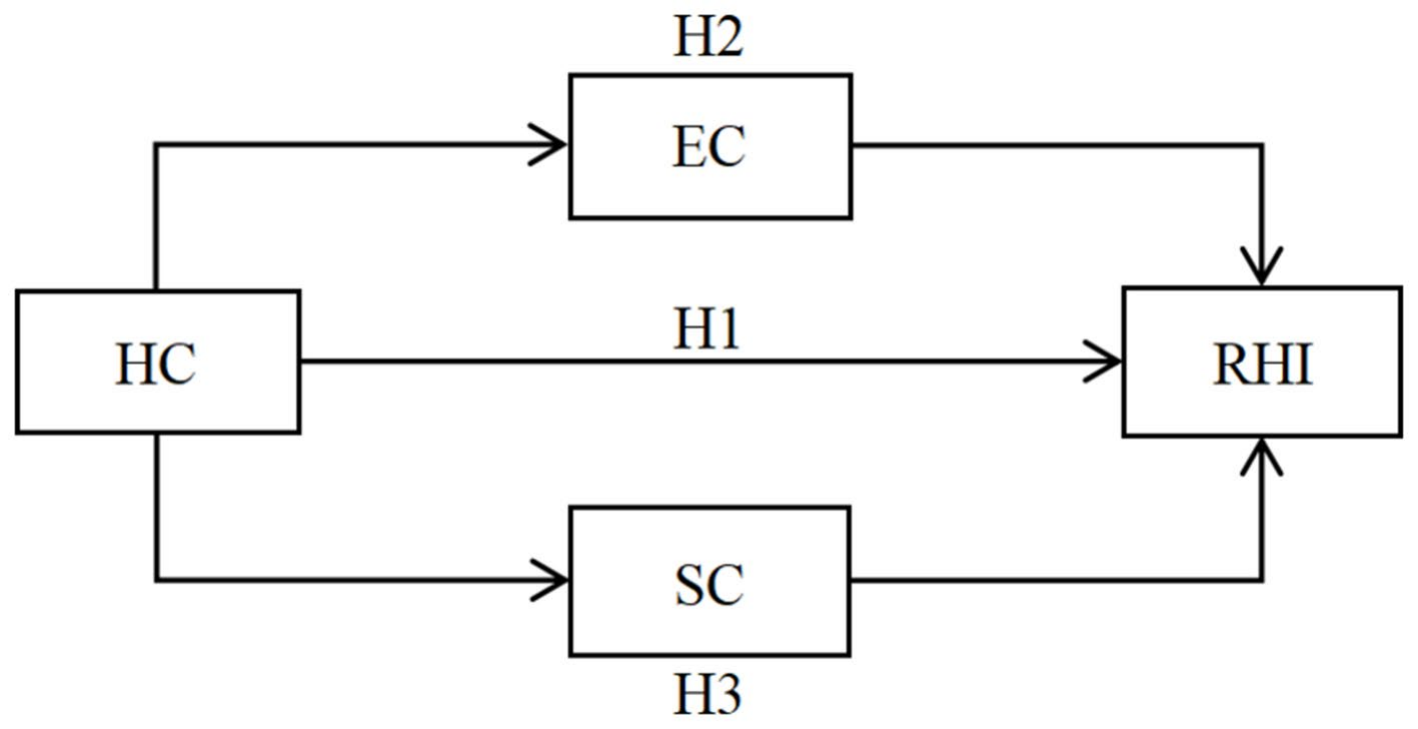
Theoretical model.

Figure 1 illustrates the theoretical model of this study.

## Methods

### Study context

The data used in this study were collected from a random anonymous questionnaire. During the survey, the respondents have known and agreed to use the data for scientific research. The random anonymous questionnaire was carried out in accordance with the “Measures for the Ethical Review of Biomedical Research Involving Humans,” published by the National Health and Family Planning Commission of the People’s Republic of China.

### Participants and survey

The subjects of this study are migrant workers who have been working in cities for more than 6 months. In terms of data acquisition, according to the principle of random sampling, Zhejiang, Guizhou, and Hunan provinces located in eastern, western, and central China were selected, respectively, and several cities in each province were selected as survey sites. We surveyed migrant workers with household registration in these areas. The random anonymous questionnaire was conducted from December 2017 to March 2018, taking advantage of the opportunity for migrant workers to return to their hometowns for the Spring Festival and adopting the method of household survey. To ensure that the questions in the questionnaires were free from any ambiguity, the instrument was piloted and tested to a cross section of the targeted population for this study. After the successful piloting and testing, migrant workers were instructed to fill out the questionnaire on a one-to-one basis. Finally, a total of 887 questionnaires were obtained, and according to the needs of this study, the invalid questionnaires and questionnaires with missing values were removed, and 810 valid questionnaires were obtained. Table 1 reports the descriptive statistics of the sample (i.e., gender, age, marriage, and education level).

**Table 1:** Sample distribution and characteristics (*n* = 810).

Demographics		Frequency	Scale (%)
Gender	Female	307	37.9
	Male	503	62.1
Age	Born before the 1970s	358	44.2
	Born in the 1970s and later	452	55.8
Marriage	Unmarried	303	37.4
	Married	507	62.6
Education level	Primary and below	123	15.2
	Junior high school degree	311	38.4
	High school degree	305	37.7
	Bachelor degree or above	71	8.7

### Measures

#### Outcome variables

The outcome variable in this study is migrant workers’ RHI, which is a subjective attitude toward returning home. The binary variable “whether you have any desire to return home” was used to measure RHI.

#### Explanatory variables

The core explanatory variable in this study is HC. Health is a multidimensional concept (44), and this study measures the HC from two dimensions of physical health and psychological health. Considering the difficulty of measuring objective health status, this study adopts self-assessed health to measure migrant workers’ HC. The variable used to assess physical health and psychological health was referred to Newbold and Danforth (45) and Xie et al. (46), “Physical health perception” and “Self-confidence in life” were used to measure physical health and mental health, respectively, and two variables were measured by a 5-point Likert scale regarding the level of agreement (1 = strongly disagree to 5 = strongly agree).

#### Mediating variables

Mediating variables in this study are the EC and SC. The variable used to assess economic capital (EC) was referred to Cheng and Lin (47), which chose “Monthly wage income” as the proxy variable for EC. The scale used to assess social capital (SC) was assessed from Li et al. (48) and Li and Mao (53), which consisted of seven items. The fuzzy comprehensive evaluation method and the entropy value method were used to calculate SC.

#### Control variables

Previous studies have shown that individual characteristics (IC) have an important impact on migrant workers’ RHI; based on this, this study refers to the relevant studies (17–19), selecting “gender,” “marriage,” “age,” and “education level” as control variables to reflect the influence of individual characteristics on migrant workers’ RHI. Family characteristics (FC) are also important factors affecting migrant workers’ RHI, and this study refers to relevant studies (2, 20) and selects “number of family members” and “family members serving in local government” as proxy variables for family human capital and social capital to be included in the model. Family migration also affects migrant workers’ RHI. Migration of migrant workers is a decision based on the principle of family maximization, and whether migrant workers go to the city alone or with family members may affect their choice; thus, this study sets two dummy variables, namely, “spouse moving with family” and “children moving with family,” which are included in the model. According to push and pull theory, regional characteristics (RC) are an important factor influencing the RHI. Thus, this study refers to Li and Qiu (49) and selects “city administrative level” and “hometown public service level” as proxies for the characteristics of the outflow place and the inflow place, respectively, to be included in the model. The definition and descriptive statistics of each variable are shown in Table 2.

**Table 2:** Variable descriptive statistics.

Variables	Label	Assignment	Mean	S.D.
Returning home intentions	RHI	No = 0, Yes = 1	0.48	0.500
Physical health	PH	Health level 1–5	3.60	0.893
Mental health	MH	Confidence level 1–5	3.05	0.730
Economic capital	EC	Monthly wage income level 1–5	2.87	0.999
Social capital	SC	Calculated from the formula	2.83	0.757
Gender	IC1	Male = 1; Female = 0	0.62	0.431
Marriage	IC2	Married = 1; Unmarried = 0	0.63	0.419
Age	IC3	Born in the 1970s and later = 1; Born before the 1970s = 0	0.55	0.498
Education level	IC4	Highest level of education 1–4	2.40	0.864
Household human capital	FC1	Number of family members level 1–4	2.46	0.998
Household social capital	FC2	Family member in local government (N = 0, Y = 1)	0.08	0.268
Spouse accompanying	FC3	Yes = 1; No = 0	0.60	0.489
Children accompanying	FC4	Yes = 1; No = 0	0.47	0.499
City administrative level	RC1	level 1–4	2.93	1.053
Hometown economic level	RC2	level 1–5	2.88	0.740

### Data analysis

In this study, Stata18.0 was used for data analysis. The probit model was used to analyze the influence of HC on RHI. Stepwise coefficient test and Sobel test were used to analyze the mediating effects of EC and SC in HC affecting RHI.

We used three methods of propensity score matching(PSM), replacing the explained variable (REV), and transforming the model (TM) for the robustness test to avoid bias caused by endogeneity issues.

### Benchmark regression model

Considering that the explained variable of RHI is a dichotomous variable, this study chooses the robust probit model for estimation. To better examine the impact of HC on RHI, this study puts the same control variables in all models. The regression model of this study is as Equation 1:


(1) $$RHI_{i}=cHC_{i}+\sum \gamma_{i}P_{i}+\sum \delta_{i}F_{i}+\sum \lambda_{i}E_{i}+\varepsilon_{i}$$

In the formula, RHI is the explained variable, and HC is the explanatory variable, including PH and MH, P , F , and E are the control variables of individual characteristics (IC), family characteristics (FC), and regional characteristics (RC), respectively, where individual characteristics include four variables of “gender,” “age,” “marriage,” and “education level,” and family characteristics include “family human capital” and “social capital” and two dummy variables: “Spouse accompanying” and “children accompanying.” Regional characteristics include the variables of “city administrative level” and “hometown economic development level.” c , γ , δ , and λ are the coefficients, and ε is the error term.

### Mediation effect test model

There are various methods to test the mediating effect, and this study refers to Wen et al. (50) and adopts the stepwise coefficient test (SCT) and Sobel test (ST) to analyze the mediating effects of EC and SC in HC affecting RHI. To estimate the parameters of HC affecting the mediating variables (EC and SC), and HC and mediating variables jointly affecting the RHI, this study further establishes the following two regression models (see Equations 2, 3) based on the benchmark return model:


(2) $$MV_{i}=aHC_{i}+\sum \gamma_{i}P_{i}+\sum \delta_{i}F_{i}+\sum \lambda_{i}E_{i}+\varepsilon_{i}$$

(3) $$RHI_{i}=bMV_{i}+c'HC_{i}+\sum \gamma_{i}P_{i}+\sum \delta_{i}F_{i}+\sum \lambda_{i}E_{i}+\varepsilon_{i}$$

In the formula, MV is the mediating variable, including both economic capital (EC) and social capital (SC). In model (2), a is the estimated coefficient of HC on the mediator variable; in model (3), b and $c^{\prime }$ are the estimated coefficients of the mediator variable and HC on the RHI, respectively, and other variables are the same as those in the benchmark regression model (1).

## Results

### Benchmark regression results

In order to be able to interpret and compare the results more intuitively, this study reports marginal effects for probit regressions; in addition, besides the probit regression results, this study also reports the OLS estimation of the linear probability model as a reference. The regression results for the benchmark regression model are shown in Table 3, where columns (1) and (2) show the regression results for the MLE estimation of the probit model and columns (3) and (4) show the regression results for the OLS estimation of the linear model.

**Table 3:** Regression results of the baseline regression model.

Variable	RHI			
	Probit		OLS	
	Model 1 (1)	Model 2 (2)	Model 3 (3)	Model 4 (4)
PH	−0.0398^**^(0.0202)		−0.0402^**^(0.0204)	
MH		−0.0512^**^(0.0247)		−0.0513^**^(0.0247)
IC	Control	Control	Control	Control
FC	Control	Control	Control	Control
RC	Control	Control	Control	Control
*p*-value	0.0011	0.0000	0.0003	0.0002
Pseudo R^2^	0.0284	0.0429	0.0386	0.0318

1. ***, **, and * are significant at 99, 95, and 90% confidence levels, respectively. 2. Due to space limitations, the regression results of control variables are not listed.

### The direct impact of HC on RHI

The estimation results of the probit model and OLS model in columns (1) and (3) of Table 3 both showed that PH has a significant and negative impact on migrant workers’ RHI (c = −0.0398, *p* < 0.05; c = −0.0402, *p* < 0.05, respectively). The estimation results of the probit model and OLS model in columns (2) and (4) of Table 3 both showed that MH has a significant and negative impact on migrant workers’ RHI (c = −0.0512, *p* < 0.05; c = −0.0513, *p* < 0.05, respectively). Therefore, H1 was supported and accepted.

### Robustness test

#### Propensity score matching

In this study, propensity score matching (PSM) was used to analyze the effect of health capital on migrant workers’ RHI, so as to control potential endogenous problems. Physical health level and mental health level were treated as processing variables, reflow intention was the result variable, and the control variable in the benchmark model was the covariate. In terms of the assignment of variables, physical health and mental health are divided into two levels according to the average level. Those less than the average level are the low level group, and the value is 0; those greater than the average level are the high level group, and the value is 1. On this basis, the samples were divided into two groups of high level of health capital and low level of health capital. Four common matching methods, namely, nearest neighbor matching, caliper matching, nearest neighbor matching in caliper, and kernel matching, were used to estimate ATT. The results in Table 4 show that the average treatment effects calculated by these four different matching methods are all significant at the significance level of 5%, indicating that even if the influence of endogenous problems is controlled, healthy capital will still significantly reduce the return intention of migrant workers in cities, which proves the robustness of the benchmark regression model.

**Table 4:** The analysis result of the PSM.

Matching method	Physical Health			Mental Health		
	ATT estimates	S.E.	T	ATT estimates	S.E.	T
Nearest neighbor matching	−0.1253	0.0545	−2.30^**^	−0.1187	0.0601	−1.98^**^
Caliper Matching	−0.1180	0.0543	−2.17^**^	−0.1159	0.0588	−1.97^*^
Nearest neighbor matching within the caliper	−0.1021	0.0464	−2.20^**^	−0.1183	0.0538	−2.20^**^
kernel matching	−0.1418	0.0641	−2.21^**^	−0.1008	0.0510	−1.97^**^

**, **, and * are significant at 99, 95, and 90% confidence levels, respectively.

#### Replacing the explained variable

Considering that there is a clear relationship between migrant workers’ RHI and urban settlement intentions (USI), this study changes the explained variable to USI for the robustness test. The probit regression analysis is carried out with USI replacing the variable of RHI in the benchmark model, which can not only verify the robustness of the model but also further judge the authenticity of the questionnaire data. Columns (3) and (4) in Table 5, respectively, list the estimated results of Probit regression. Column (5) is the effect of migrant workers’ physical health on their USI. Column (6) is the effect of migrant workers’ mental health on their USI. The results show that the significance level of the core explanatory variables “physical health” and “mental health” is basically consistent with the benchmark model, and both are significant at the level of 5%, while the coefficient symbol is opposite to the benchmark model, which also verifies that when migrant workers are physically and mentally healthy, they are more willing to stay in the city and settle down, rather than choose to return. It also proves that the regression results of the benchmark model are robust.

**Table 5:** Regression results of the REV.

Variable	RHI		USI	
	Model 1 (1)	Model 2 (2)	Model 5 (3)	Model 6 (4)
PH	−0.0398**(0.0202)		0.0385**(0.1907)	
MH		−0.0512**(0.0247)		0.0524**(0.0233)
IC	Control	Control	Control	Control
FC	Control	Control	Control	Control
RC	Control	Control	Control	Control
P-value	0.0011	0.0000	0.0043	0.0025
Pseudo R^2^	0.0284	0.0429	0.0386	0.0318

1. ***, **, and * are significant at 99, 95, and 90% confidence levels, respectively. 2. Due to space limitation, the regression results of control variables are not listed.

#### Transforming the model

This study further transformed the model to conduct a robustness test and selected a logit regression model and linear regression model to test the relationship between HC and RHI. Table 6 shows the regression results. Columns (1) and (2) in Table 6, respectively, list the estimated results of linear regression. Columns (3) and (4) in Table 6, respectively, list the estimated results of logit regression. Column (3) is tvhe effect of migrant workers’ physical health on their RHI, and column (4) is the effect of migrant workers’ mental health on their RHI. The results show that the significance level of the core explanatory variables “physical health” and “mental health” is basically consistent with the benchmark model, and both are significant at the level of 5%, which also proves that the regression results of the benchmark model are robust.

**Table 6:** Regression results of the TM.

Variable	RHI			
	OLS		Logit	
	Model 3 (1)	Model 4 (2)	Model 7 (3)	Model 8 (4)
PH	−0.0402^**^(0.0204)		−0.0399**(0.0203)	
MH		−0.0513^**^(0.0247)		−0.0512**(0.0250)
IC	Control	Control	Control	Control
FC	Control	Control	Control	Control
RC	Control	Control	Control	Control
P-value	0.0003	0.0002	0.0016	0.0003
Pseudo R^2^	0.0386	0.0318	0.0284	0.0317

1. ***, **, and * are significant at 99, 95, and 90% confidence levels, respectively. 2. Due to space limitations, the regression results of control variables are not listed.

### Regional heterogeneity analysis

The subjects of this study are from Hunan, Guizhou, and Zhejiang provinces, which are located in central, western, and eastern China, respectively. Considering the differences in regional economic and social development in China, the effect of migrant workers’ HC on their RHI may vary regionally. In this study, all the research samples were divided into eastern, central, and western regions for re-empirical analysis.

The results of Table 7 showed that the relationship between HC and RHI varied in western, central, and eastern China. The HC of migrant workers in western China has a significant hindering effect on their RHI. In the regression model of the western region, the effects of PH and MH on RHI are significantly negative at 1 and 10%, respectively, indicating that physical health and mental health have negative effects on migrant workers’ RHI. The HC of migrant workers in central China has a significant hindering effect on their RHI. In the regression model of the central region, the effects of PH and MH on RHI are significantly negative at 10 and 5%, respectively, indicating that physical health and mental health have negative effects on migrant workers’ RHI. In conclusion, the effect of HC on the RHI of migrant workers in central and western China is consistent with the results of the benchmark model.

**Table 7:** Regression results of the subregional regression model.

Variable	RHI					
	Western		Central		Eastern	
	Model 9 (1)	Model 10 (2)	Model 11 (3)	Model 12 (4)	Model 13 (5)	Model 14 (6)
PH	−0.1403^***^(0.0161)		−0.0469^*^(0.0264)		0.0566 (0.0377)	
MH		−0.0443^*^(0.0229)		−0.0782^**^(0.0375)		−0.0302(0.0434)
IC	Control	Control	Control	Control	Control	Control
FC	Control	Control	Control	Control	Control	Control
RC	Control	Control	Control	Control	Control	Control
P-value	0.0065	0.0392	0.0074	0.0047	0.5647	0.6954
Pseudo R^2^	0.1260	0.0959	0.0481	0.0503	0.0269	0.0230

1. ***, **, and * are significant at 99, 95, and 90% confidence levels, respectively. 2. Due to space limitations, the regression results of control variables are not listed.

The effect of HC on the RHI of migrant workers in eastern China was not significant, which may be related to the level of economic development in eastern China. The eastern region is the region with the highest level of economic development in China, which makes the region rich in job opportunities. No matter from the import or export of migrant workers, the eastern region is the main settlement of migrant workers, other conditions remain unchanged, and the farmers’ trade unions give priority to employment in the eastern region. Based on this, migrant workers in the eastern region are generally employed in cities and towns in the eastern region, they are employed nearby, and the work site is very close to their hometown. Therefore, they can often go back to their hometown. Working nearby also helps migrant workers take care of family members living in their hometowns. All these may affect the relationship between HC and RHI.

### Mediation analysis

The regression results of the benchmark model show that both PH and MH have a significant effect on migrant workers’ RHI. To further understand the mediating effects of EC and SC in HC affecting RHI, this study continues to estimate Model 2 and Model 3.

### Mediating effect test of EC

The results of column (1) in Table 8 showed that PH has a significant and positive impact on EC (*a =* 0.0911, *p <* 0.05); the results of column (2) show that EC has a significant and negative impact on migrant workers’ RHI (*b = −*0.0656, *p <* 0.05); therefore, the mediation effect of EC between PH and RHI is significant. In addition, since the effect of PH on migrant workers’ RHI in column (2) is also significant at a 10% level (c` ≠ 0), therefore, the mediating effect of EC between PH and RHI is partially mediated, and the ratio of the mediating effect to the total effect is *ab/c =* 15.02%. Through further Sobel test, the calculated Z_Sobel_ value is greater than 1.96 (Z_Sobel_ = 2.0349), which indicates that the mediation effect of EC between PH and RHI is significant using the Sobel test (ST). The result of column (3) in Table 8 showed that MH has a significant and positive impact on EC (*a =* 0.2503, *p <* 0.05); the result of column (4) shows that EC has a significant and negative impact on migrant workers’ RHI (*b = −*0.0629, *p <* 0.05); therefore, the mediation effect of EC between MH and RHI is significant. In addition, since the effect of MH on migrant workers’ RHI in column (4) is not significant (c` = 0), therefore, the mediating effect of EC between MH and RHI is fully mediated, and the ratio of the mediating effect to the total effect is *ab/c =* 39.55%. Through further Sobel test, the calculated Z_Sobel_ value is greater than 1.96 (Z_Sobel_ = 2.8049), which indicates that the mediation effect of EC between MH and RHI is significant using the Sobel test (ST).

**Table 8:** Results of the mediating effect test for economic capital.

Variable	PH		MH	
	Model 15 (1)	Model 16 (2)	Model 17 (3)	Model 18 (4)
EC		−0.0656^***^(0.0181)		−0.0629^***^(0.0181)
PH	0.0911^**^(0.0370)	−0.0333^*^(0.0195)		
MH			0.2503^***^(0.0525)	−0.0296(0.0252)
IC	Control	Control	Control	Control
FC	Control	Control	Control	Control
RC	Control	Control	Control	Control
P-value	0.0000	0.0000	0.0000	0.0000
Pseudo R^2^	0.0991	0.0420	0.1233	0.0438
Z_Sobel_	2.0349		2.8049	

1. ***, **, and * are significant at 99, 95, and 90% confidence levels, respectively. 2. Due to space limitations, the regression results of control variables are not listed.

Combining the results of the two methods SCT and ST, the mediation effect of EC between HC and RHI is significant, which means H2 was supported and accepted.

### Mediating effect test of SC

The results of column (1) in Table 9 showed that the effect of PH on SC is insignificant (*a =* 0.0428, *p >* 0.10), and the results of column (2) in Table 9 showed that the effect of SC on RHI is also insignificant (*b = −*0.0101, *p >* 0.10), which indicates that the mediation effect of SC between PH and RHI is insignificant using a stepwise coefficient test (SCT). Through further Sobel test, the calculated Z_Sobel_ value is less than 1.96 (Z_Sobel_ = 1.7764), which also indicates that the mediation effect of SC between PH and RHI is insignificant using the Sobel test. Therefore, the mediation effect of SC between PH and RHI is insignificant. The results of column (3) in Table 9 showed that the effect of MH on SC is significant (*a =* 0.3372, *p <* 0.05), and the results of column (4) in Table 9 showed that the effect of SC on RHI is insignificant (*b = −*0.0271, *p >* 0.10), which indicates that the mediation effect of SC between MH and RHI is insignificant using a stepwise coefficient test (SCT). Through further Sobel test, the calculated Z_Sobel_ value is less than 1.96 (Z_Sobel_ = 0.3841), which indicates that the mediation effect of SC between MH and RHI is insignificant using the Sobel test. Therefore, the mediation effect of SC between MH and RHI is also insignificant.

**Table 9:** Results of the mediating effect test for social capital.

Variable	PH		MH	
	Model 19 (1)	Model 20 (2)	Model 21 (3)	Model 22 (4)
SC		−0.0101(0.0251)		−0.0271(0.0262)
PH	0.0428 (0.0302)	−0.0402^**^(0.0203)		
MH			0.3372^***^(0.0393)	−0.0606^**^(0.0261)
IC	Control	Control	Control	Control
FC	Control	Control	Control	Control
RC	Control	Control	Control	Control
P-value	0.0000	0.0019	0.0000	0.0002
Pseudo R^2^	0.0452	0.0286	0.1649	0.0326
Z_Sobel_	1.7764		0.3841	

1. ***, **, and * are significant at 99, 95, and 90% confidence levels, respectively. 2. Due to space limitations, the regression results of control variables are not listed.

Combining the results of the two methods SCT and ST, the mediation effect of SC between HC and RHI is also insignificant, which means H3 was not supported. The group of migrant workers has a rural social network derived from rural life and an urban social network derived from urban work. The two kinds of social capital have different effects on the RHI of migrant workers. Rural social capital can promote RHI, while urban social capital can hinder RHI. This may be the result that the mediating effect of social capital in HC’s influence on RHI is not significant.

## Discussion and conclusion

### Discussion

The primary aim of this study is to help understand the influence of migrant workers’ HC on their RHI. For this, we developed an empirical model of migration intentions to probe whether migrant workers’ HC can affect their RHI and analyze the paths that this influence may follow to form migrant workers’ RHI. This study reveals that migrant workers’ HC has a significant direct negative impact on their RHI. After controlling for endogenous issues, the results remained robust. The result supports the Salmon Bias Hypothesis, which holds that immigrants who are unhealthy or whose health deteriorates are more likely to return to or be closer to their communities of origin than those who are healthy (5, 25). The research conclusion is also consistent with the study of Shang et al. (27) and Huang and Fang (22), who found that Chinese rural laborers with poor health conditions had a relatively high willingness to return.

This study further found that mental health has a greater impact on migrant workers’ RHI than physical health, which is very important, because most of the previous studies measure health capital from a single dimension of physical health, and the conclusion of this study indicates that previous studies may underestimate the impact of health capital on migrant workers’ RHI. In the study of regional heterogeneity, this study found that the influence of HC on the RHI of migrant workers in central and western China was significantly negative, but this relationship was not significant in eastern China. The results show that there is regional heterogeneity in the effect of HC on the RHI of migrant workers in China. In the study of the mediating effect, the study found that HC has a positive and significant indirect impact on RHI through economic capital. However, the mediating role of social capital in the relationship between HC and RHI is not significant, which is inconsistent with the research of Ehsan (42) and Ling and Liu (43).

#### Contribution to the literature and theoretical implications

This study extends and deepens our understanding of the formation of returning home intentions from a health capital perspective and adds to the literature by focusing on both physical and mental health dimensions of the pre-action phase in the migration process. This study reveals that migrant workers’ health capital has a significant impact on their returning home intentions. Migrant workers’ health capital is important in improving their economic capital, thereby restricting the formation of their returning home intentions. This study confirms the validity of the “salmon bias effect” hypothesis in the Chinese context, which is consistent with the results of previous studies on health and migration, and provides an empirical case for this hypothesis in the Chinese context. This study further deepens the understanding of the concept of health capital from the two dimensions of physical health and mental health; in contrast, previous studies focused on the impact of health capital on migrant workers’ returning home intentions from a single dimension of physical health.

#### Practical implications

In addition to the theoretical significance, this study also has a certain practical significance for the development and promotion of migrant workers’ health capital and migratory behavior. The results of this study show that migrant workers with poor health capital are more likely to return, which partly explains why the implementation of China’s rural revitalization strategy is not ideal. The reason is that migrant workers with high health capital are more inclined to stay in the city, while those with unsatisfactory health capital are more likely to choose backflow. This backflow is a passive backflow, which has limited driving effect on rural economic development. Therefore, the conclusion of this study can provide theoretical support for the government to evaluate the effect of migrant workers’ return in rural economic development. To improve the health capital of returning migrant workers, rural local government could use the model to improve returning migrant workers’ health capital from both physical and psychological dimensions. Moreover, under the current dual-strategy background of China’s new-type urbanization and rural revitalization strategy, to promote the integrated development of urban and rural areas, the national government could use the model to introduce policies that helps to promote the bidirectional flow of migrant workers between urban and rural areas.

#### Limitations and future research

The present study is limited in at least three ways. First, this study adopts the subjective evaluation method to measure the health capital and uses two proxy variables of “physical health status” and “life confidence level” to measure the physical health and mental health of migrant workers, respectively. In future studies, mature scales can be used to measure health capital, and the reliability of the results may be better. Second, this study focuses on the mediating effect of economic capital and social capital between healthy capital and returning home intention, but there may be other variables, such as the capacity dimension of human capital and cultural capital, and the mediating effect of these factors can be discussed in future studies.

## Data Availability

The raw data supporting the conclusions of this article will be made available by the authors, without undue reservation.
